# Development of serum-free and grain-derived-nutrient-free medium using microalga-derived nutrients and mammalian cell-secreted growth factors for sustainable cultured meat production

**DOI:** 10.1038/s41598-023-27629-w

**Published:** 2023-01-10

**Authors:** Kumiko Yamanaka, Yuji Haraguchi, Hironobu Takahashi, Ikko Kawashima, Tatsuya Shimizu

**Affiliations:** 1 Institute of Advanced Biomedical Engineering and Science, Tokyo Women’s Medical University, 8-1 Kawada-cho, Shinjuku-ku, Tokyo, 162-8666 Japan; 2 IntegriCulture Inc., The Advanced Technology Research Laboratory, Tokyo Women’s Medical University, TWIns N101, 8-1 Kawada-cho, Shinjuku-ku, Tokyo, 162-8666 Japan

**Keywords:** Cellular microbiology, Cell growth, Biotechnology

## Abstract

Considering the amount of global resources and energy consumed, and animal welfare issues associated with traditional meat production, cultured meat production has been proposed as a solution to these problems and is attracting worldwide attention. Cultured meat is produced by culturing/proliferating animal muscle cells in vitro. This process requires significant amounts of culture medium, which accounts to a major portion of the production cost. Furthermore, it is composed of nutrients derived from grains and heterotrophic microorganisms and fetal bovine serum (FBS), which will impact the sustainability of cultured meat in future. Here, we developed a novel medium containing nutrients extracted from microalga and cell-secreted growth factors. First, rat liver epithelial RL34 cells were cultured by adding *Chlorella vulgaris* extract (CVE) to inorganic salt solution. The supernatant, containing the RL34 cell-secreted growth factors, was used as the conditioned medium (CM). This CM, with CVE added as a nutrient source, was applied to primary bovine myoblast cultures. This serum-free and grain-derived-nutrient-free medium promoted the proliferation of bovine myoblasts, the main cell source for cultured beef. Our findings will allow us to take a major step toward reducing production costs and environmental impacts, leading to an expansion of the cultured meat market.

## Introduction

Animal cells are generally cultured in basal media supplemented with fetal bovine serum (FBS) which provides the various factors essential for cell growth. The basal media contains nutrients (glucose, amino acids, vitamins, etc.) that are usually derived from grains or heterotrophic microorganisms, and inorganic salts (sodium, potassium, calcium, etc.). Regarding the major raw materials used, grain production is greatly affected by climate change^1^. The employment of chemical fertilizers and pesticides can cause environmental pollution^2^. Moreover, the usage of FBS, being of animal origin, poses animal welfare issues as well as contamination risks from pathogens. An unstable supply and high costs are also important concerns^3–5^. The issues with culture medium are inescapable in cultured meat production.

Compared to grains, microalgae have higher growth capacity and usable protein content in their biomass. Since microalgae can be cultured on land that is unsuitable for agriculture crops, it does not compete with traditional agriculture. Moreover, the high CO_2_-fixing ability of microalgae can aid in achieving carbon neutrality. Recently, microalgae have been attracting attention as a substitute for fossil fuels and as an edible protein. Culturing microalgae in industrial wastewater provides the dual benefits of nutrient removal which prevents eutrophication as well as low-cost biomass production^6^. In addition, we reported that it is possible to cultivate microalgae using animal cell waste medium^7^. Tuomisto et al. examined the environmental impacts of meat production using the life cycle assessment method and reported that producing cultured meat using microalgal nutrient sources has a drastically lower environmental impact compared to conventional meat production^8^. Thus, microalga is an optimal cell source to efficiently provide nutrients to animal muscle cells.

**Figure 1:**
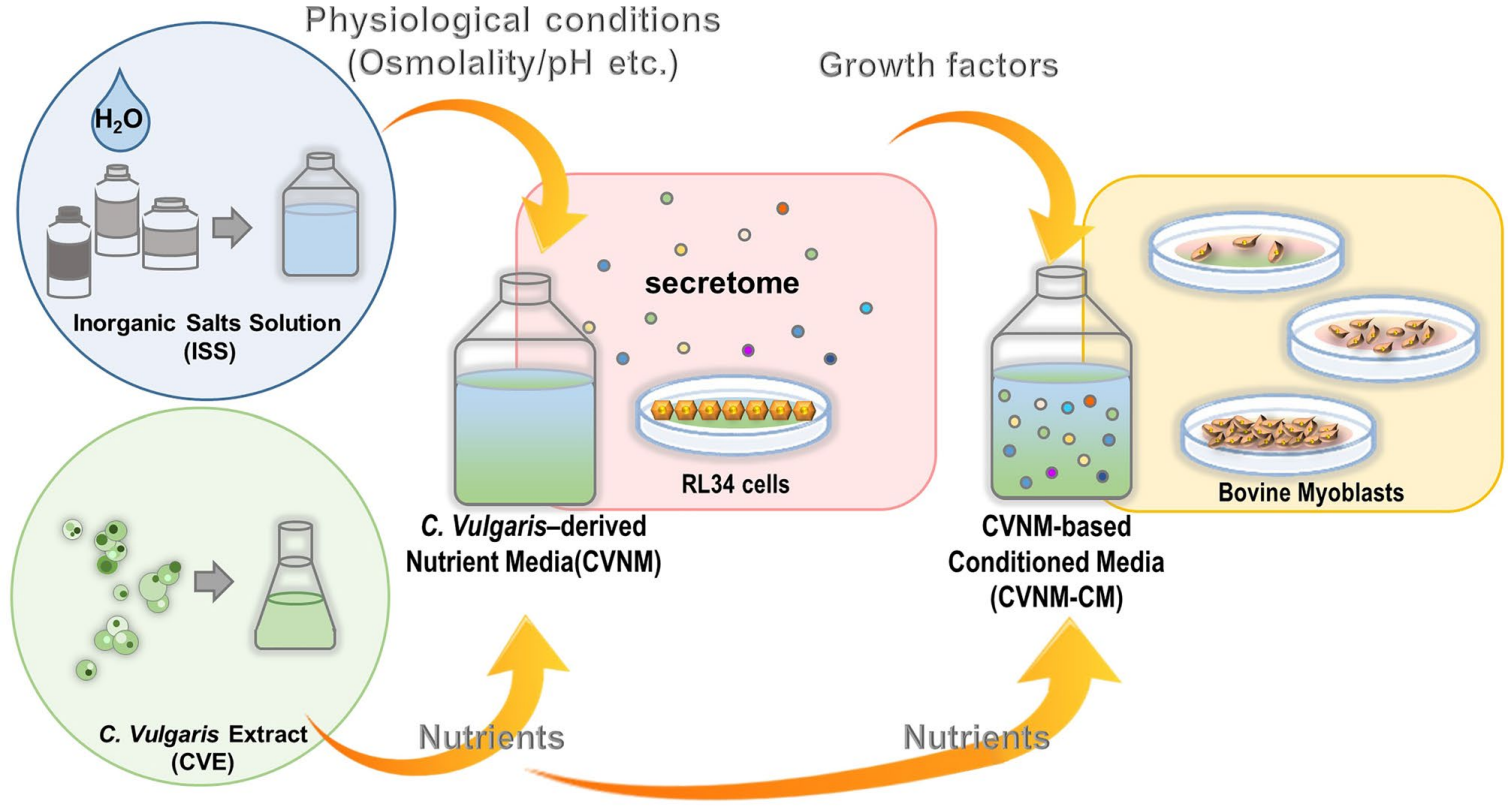
Illustration of the procedures for media preparation. *Chlorella vulgaris*-derived nutrient media (CVNM) are formulated by adding *Chlorella vulgaris* extract (CVE) to inorganic salt solution (ISS) as a nutrient source for maintaining RL34 cells. The secretome containing growth factors is secreted by RL34 cells into the culture supernatant. The supernatant containing the growth factors is known as conditioned medium (CM). The CVNM-based conditioned media (CVNM-CM), with CVE added to restore the nutrients consumed by the RL34 cells, are provided to bovine myoblasts.

We previously reported the successful culture of myoblasts using microalgal extract as a nutrient source^9,10^. The culture supernatant of animal cells, also known as ‘conditioned medium’ (CM), contains various factors secreted by the cells themselves, such as growth factors and cytokines. We hypothesized that by selecting optimal animal cells and using their CM to culture animal myoblasts, the cells could proliferate without the need for animal sera. Here, we propose a new approach wherein the nutrients in the culture medium are provided by a microalgae extract, and the FBS is replaced by growth factors in the CM. Figure 1 illustrates the preparation process of our serum-free and grain-derived-nutrient-free medium.

In this study, we provide evidence for the following: (1) rat liver epithelial RL34 cells are ideal for use as provider cells (named because they ‘provide’ growth factors) for the proliferation of bovine myoblasts, which are the main cell source of cultured beef; (2) *Chlorella vulgaris* (*C. vulgaris*), a highly nutritious microalga, can be used as a nutrient source for bovine myoblasts and RL34 cells; and (3) RL34 cells could be efficiently cultured by combining optimized acid hydrolysis and ultrasonic fractionation as a nutrient extraction method. This novel culture system that is independent of grain-derived nutrients and animal sera will greatly contribute to a sustainable and low-cost cultured meat production.

## Results

### Nutrient extraction from *C. vulgaris* and its characterization

**Figure 2:**
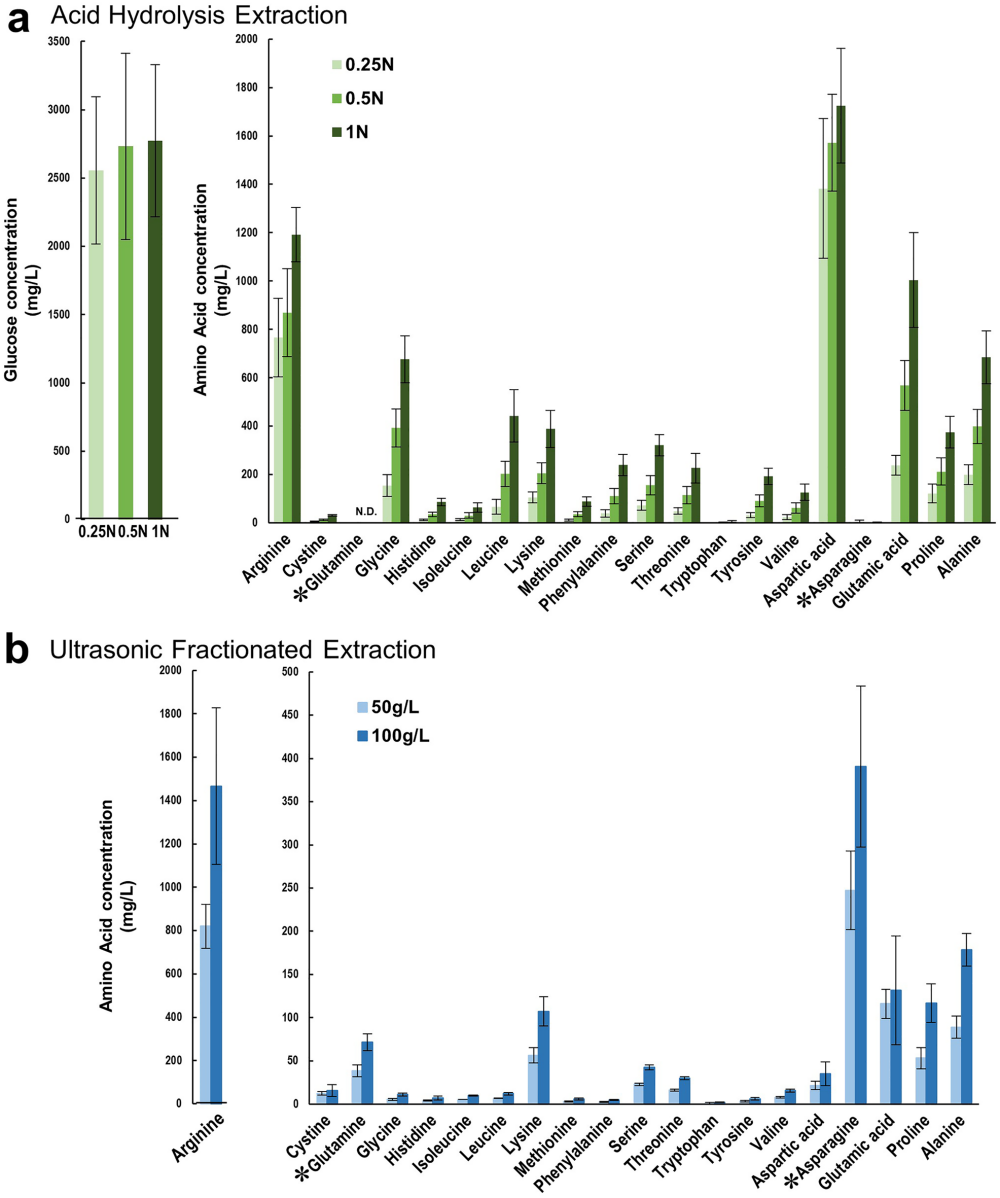
Nutrient extraction from microalgae. (**a**) Acid hydrolysis extraction was performed by hydrolyzing powdered *Chlorella vulgaris* with hydrochloric acid of different normalities (0.25, 0.5, and 1 N), reaction temperature of 100 ℃, and reaction time of 24 h (**b**) Ultrasonic fractionated extraction was performed by subjecting different concentrations of *C. vulgaris* (dry weight: 50 g/L, 100 g/L dissolved in water) to ultrasonic fractionation. *Indicates the amino acids that were mostly absent in the acid hydrolysis extract but present in the ultrasonic fractionated extract. Data are presented as the mean ± standard deviation (n = 3–6).

*C. vulgaris* extract (CVE) was obtained by two preparation methods: acid hydrolysis, in which dried *C. vulgaris* powder was subjected to hydrolysis using three different normalities (N) of hydrochloric acid (0.25, 0.5, and 1 N), and ultrasonic fractionation, in which two different concentrations of *C. vulgaris* powder (50 g/L and 100 g/L dissolved in purified water) were subjected to ultrasonication. Figure 2 shows the nutrient yield of each extract. In the acid hydrolysis extract (AHE), the concentration of amino acids increased with increasing normality of hydrochloric acid, whereas that of glucose was almost similar for all the concentrations. The amino acid glutamine, which is important for mammalian cell culture and is most abundant in Dulbecco's modified Eagle's medium (DMEM), was not detected in the AHE. However, while glucose was below the detection limit, glutamine could be detected in the ultrasonic fractionated extract (UFE). Amino acid yields were high in the 100 g/L UFE; therefore, this concentration was used for subsequent experiments.

### RL34 cell culture using *C. vulgaris*-derived nutrient medium (CVNM)

**Figure 3:**
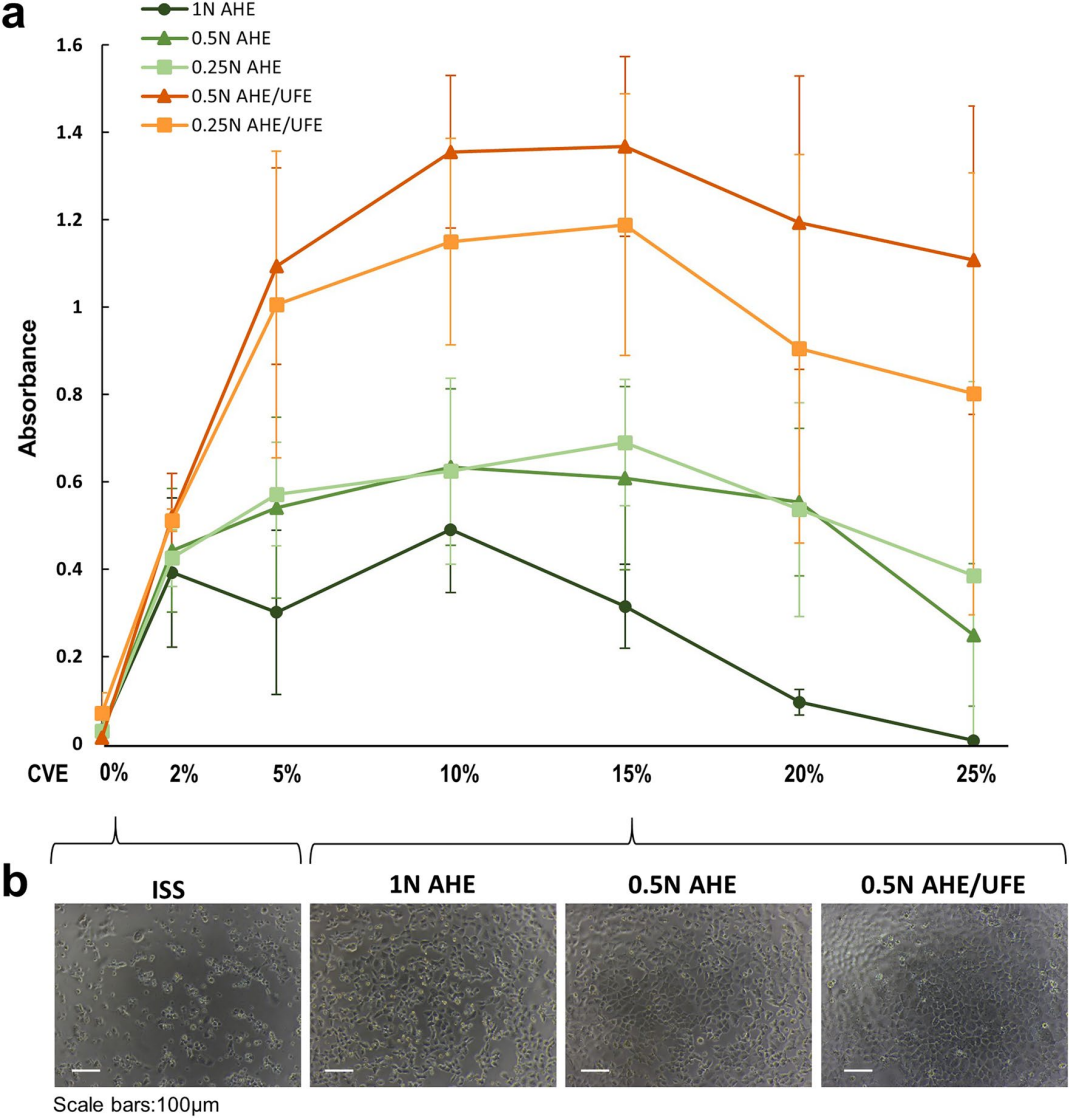
Viability of RL34 cells cultured under different CVNM conditions. Different CVNM combinations were formulated by adding between 2 and 25% CVE to ISS. (**a**) Cell viability was assessed by XTT assay. Data are presented as the mean ± standard deviation (n = 3–5). (**b**) Images of RL34 cells cultured with ISS or 15% CVNM (ISS containing 15% CVE obtained using different extraction methods; 3 representative CVEs: 1 N AHE, 0.5 N AHE, 0.5 N AHE/UFE) for 24 h. AHE/UFE: AHE and UFE mixed at a ratio of 1:1. AHE, acid hydrolysis extract; CVE, *Chlorella vulgaris* extract; CVNM, *C. vulgaris*-derived nutrient media; ISS, inorganic salt solution; UFE, ultrasonic fractionated extract.

Since CVE was previously shown to replace nutrients in DMEM^9,10^, a new basic medium was prepared by adding CVE to inorganic salt solution (ISS). The composition, osmolality, and pH of the ISS were adjusted according to those in DMEM (Table 1). When RL34 cells were cultured in ISS alone, almost all cells died within 24 h; however, adding CVE prevented cell death (Fig. 3a,b). Increasing the amount of CVE improved RL34 cell viability, which peaked at a CVE concentration of 10–15% and then tended to decreased. The survival rate was higher with 0.5 and 0.25 N AHE than with 1 N AHE. Furthermore, the addition of UFE dramatically increased the survival rate. The CVNM containing both AHE (0.5 N) and UFE showed the highest RL34 cell viability.

**Table 1:** Compositions of inorganic salt solution (ISS).

Components		Final concentration (mg/L)
Calcium chloride	CaCl_2_	200
Potassium chloride	KCl	400
Magnesium sulfate	MgSO_4_	97.67
Sodium bicarbonate	NaHCO_3_	3700
Sodium dihydrogenphosphate	NaH_2_PO_4_	108.7
Iron(III) nitrate enneahydrate	Fe(NO_3_)_3_ 9H_2_O	0.1
Phenol red	C_19_H_14_O_5_S	15
Sodium chloride	NaCl	0–6400^a^

^a^The sodium chloride concentration was modified by the final osmolality of the CVNM.

### Adding UFE prevents ‘amino acid imbalance’

Glutamine was not detected in the AHE, most likely because it was oxidized to glutamate. Similarly, the low detection of asparagine may be due to its conversion to aspartic acid. In contrast, the UFE contained both glutamine and asparagine. Although glutamine can be substituted by glutamate in C2C12 cell cultures^9^, we hypothesized that the addition of UFE could increase the survival rate of RL34 cells by compensating for the shortage of the amino acid in AHE.

**Figure 4:**
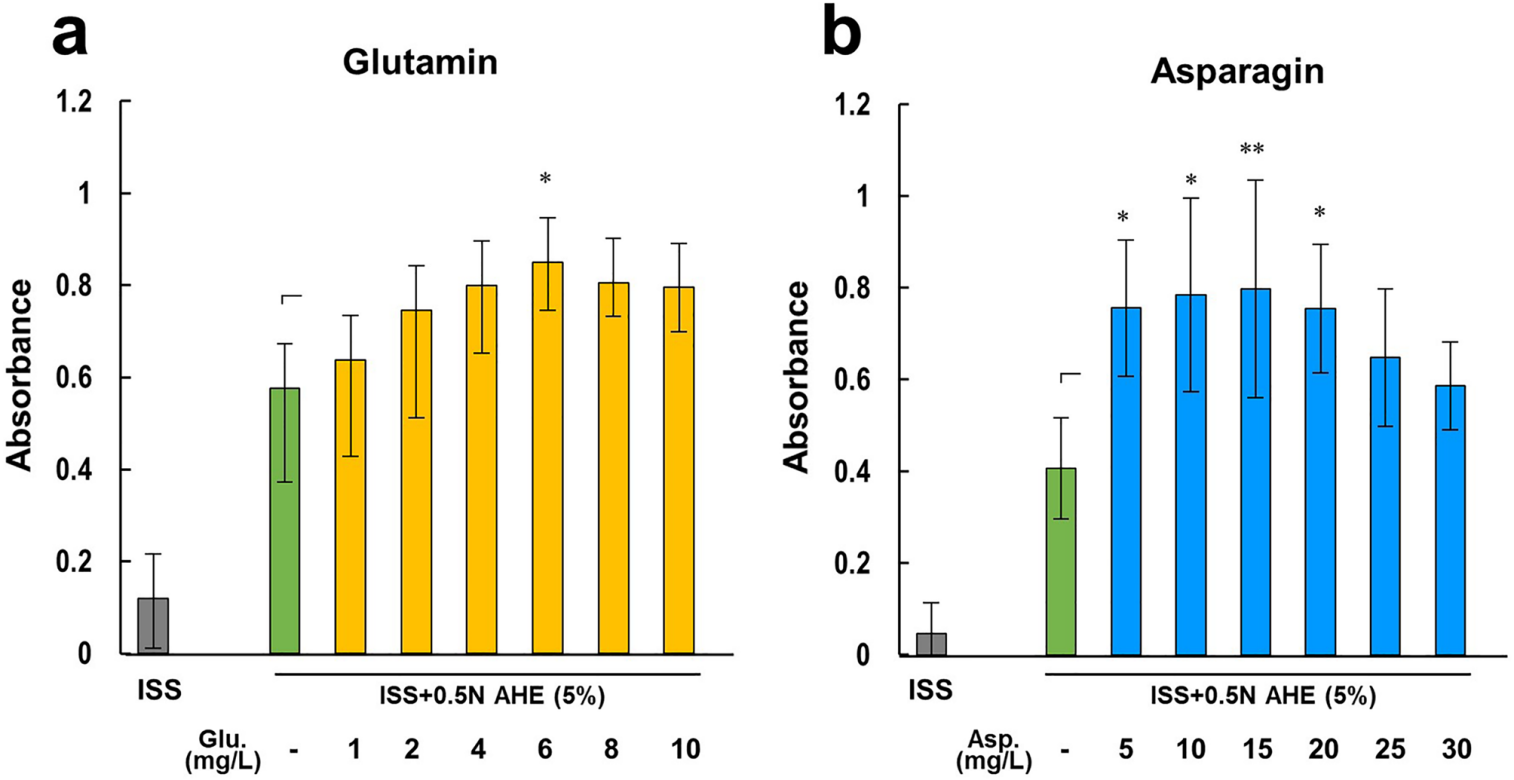
Glutamine and asparagine supplementation improved cell viability. RL34 cells were cultured in ISS containing 5% of 0.5 N AHE. Cell metabolic activity was measured after the addition of glutamine (**a**) or asparagine (**b**) in different doses. Data are presented as the mean ± standard deviation (n = 3–6). **P < 0.01, *P < 0.05. *AHE* acid hydrolysis extract, *ISS* inorganic salt solution.

To examine whether the addition of amino acids increased cell viability, RL34 cells were cultured in ISS containing 5% of 0.5 N AHE with/without the addition of glutamine or asparagine and assayed for viability after 24 h. The addition of 6 mg/L of glutamine and 5–20 mg/L of asparagine showed the highest cell viabilities (Fig. 4a,b). In both cases, higher addition of amino acids reduced cell viability. These findings suggest that adding UFE compensates for the amino acid shortfall in the AHE and that balancing the amino acid content in CVNM improves RL34 cell viability.

### Optimal pH and osmolality of RL34 cell culture under serum-free conditions

**Figure 5:**
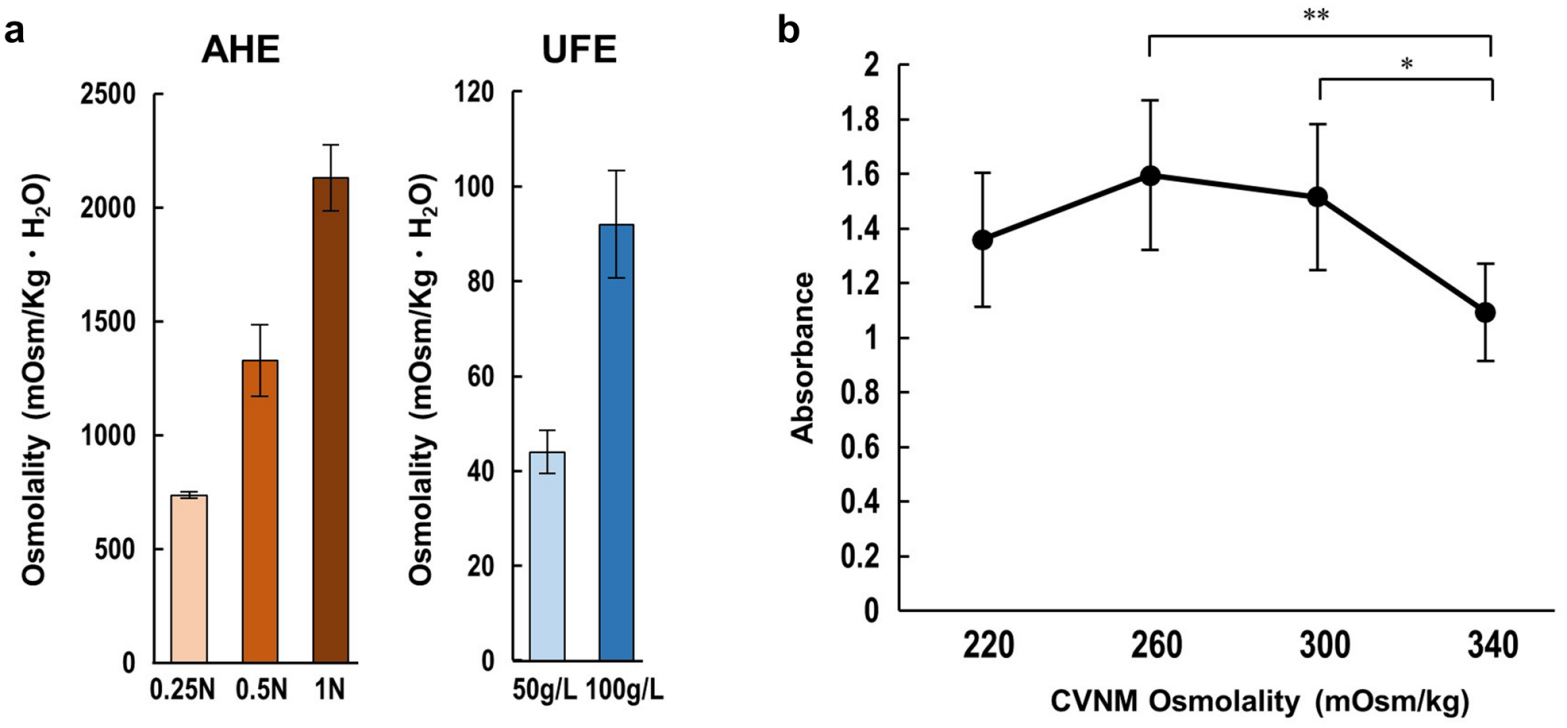
Effects of changes in osmolality on RL34 cell viability. (**a**) Osmolality of the different concentrations of CVE. Data are presented as the mean ± standard deviation (n = 6–10). (**b**) Cell viability of RL34 cell cultured under ISS containing 15% CVE (0.5 N AHE and UFE mixed at 1:1; 15% CVNM) at osmolalities of 220, 260, 300, and 340 mOsm/kg. Data are presented as the mean ± standard deviation (n = 7). **P < 0.01, *P < 0.05. AHE, acid hydrolysis extract; CVE, *Chlorella vulgaris* extract; CVNM, *C. vulgaris*-derived nutrient media; ISS, inorganic salt solution; UFE, ultrasonic fractionated extract.

Because serum plays various roles in the culture medium, cells are strongly affected by the physicochemical environment when cultured in serum-free conditions^11^. The pH was optimized at both the neutralization step of acid hydrolysis and during CVNM preparation. The pH of DMEM and CVNM were 7.82 ± 0.05 and 7.70 ± 0.10, respectively [n = 12, mean ± standard deviation (SD)]. In terms of osmolality, acid hydrolysis produces sodium chloride through the neutralization reaction, which increases the osmolality of the CVE. Therefore, it was necessary to keep the final osmolality of the CVNM constant when different percentages of CVE were added to avoid the influence of osmolality on cell viability (Fig. 3). Further, it was imperative to clarify the appropriate osmolality for adapting CVNM to RL34 cells, since high osmolality reduces cell viability^12^. The osmolality of each CVE is shown in Fig. 5a. As previously mentioned, since the neutralization reaction between acid and alkali produced sodium chloride in the AHE, the osmolality increase depended on the hydrochloric acid concentration. In contrast, as the solvent in the UFE was water, increasing the solute concentration increased the osmolality. CVNM was prepared by adding CVE (a mixture of equal volumes of 0.5 N AHE and UFE) to ISS at a concentration of 15% (15% CVNM). RL34 cells were cultured in this media with the osmolality adjusted to 220, 260, 300, and 340 mOsm/kg using sodium chloride solution, and the subsequent viability was assessed by XTT assay. The highest survival rate was observed in the 260–300 mOsm/kg media, followed by 220 and 340 mOsm/kg media (Fig. 5b). The medium with an osmolality equivalent to DMEM (340 mOsm/kg) had the lowest survival rate. We finalized the osmolality of CVNM at 260 mOsm/kg henceforth.

### Continuous cultivation of RL34 cells under serum-free conditions

**Figure 6:**
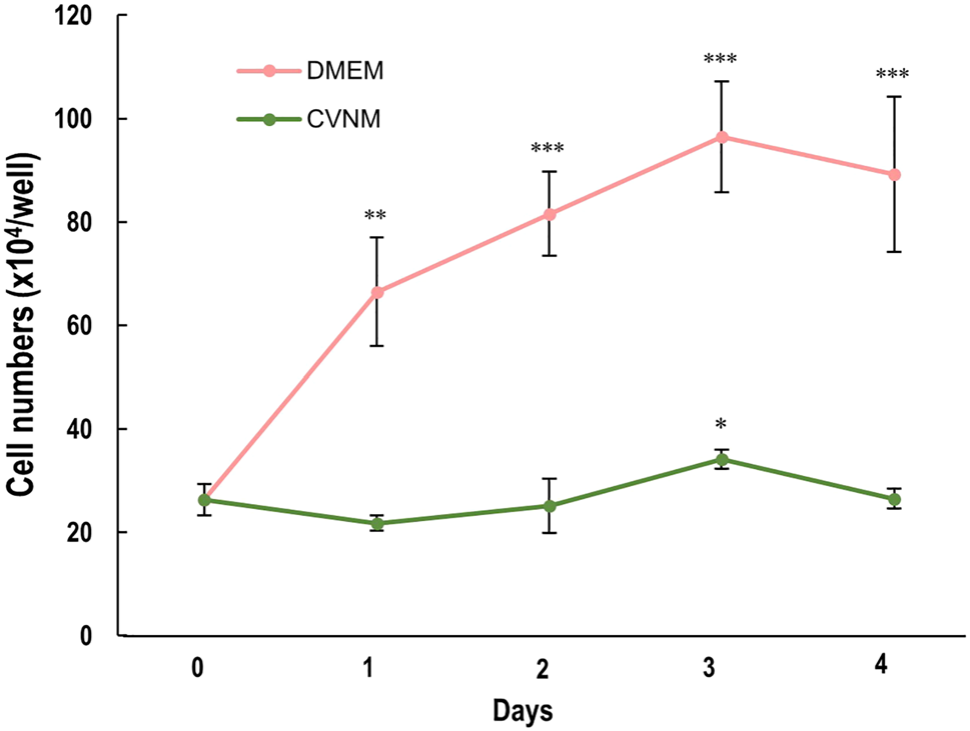
Numbers of RL34 cells cultured in a 24-well plate with DMEM or 15% CVNM for 4 days. Data are presented as the mean ± standard deviation (n = 3). ***P < 0.001, **P < 0.01, *P < 0.05. CVNM, *Chlorella vulgaris*-derived nutrient media; DMEM, Dulbecco's modified Eagle's medium.

To confirm the effect of CVNM on cell numbers over time under serum-free conditions, RL34 cells were cultured in 15% CVNM or DMEM for 4 d, with the media renewed on a daily basis, following which the cell number was counted (Fig. 6). The findings showed that while the cell count tended to decrease slightly after 24 h in the 15% CVNM, it increased and peaked on day 3. Similarly, the cell numbers kept increasing even in the serum-free conditions in the DMEM. RL34 cells proliferated better in DMEM than in 15% CVNM, possibly due to differences in nutrient content.

### Culture of bovine myogenic cells using CVNM-CM

**Figure 7:**
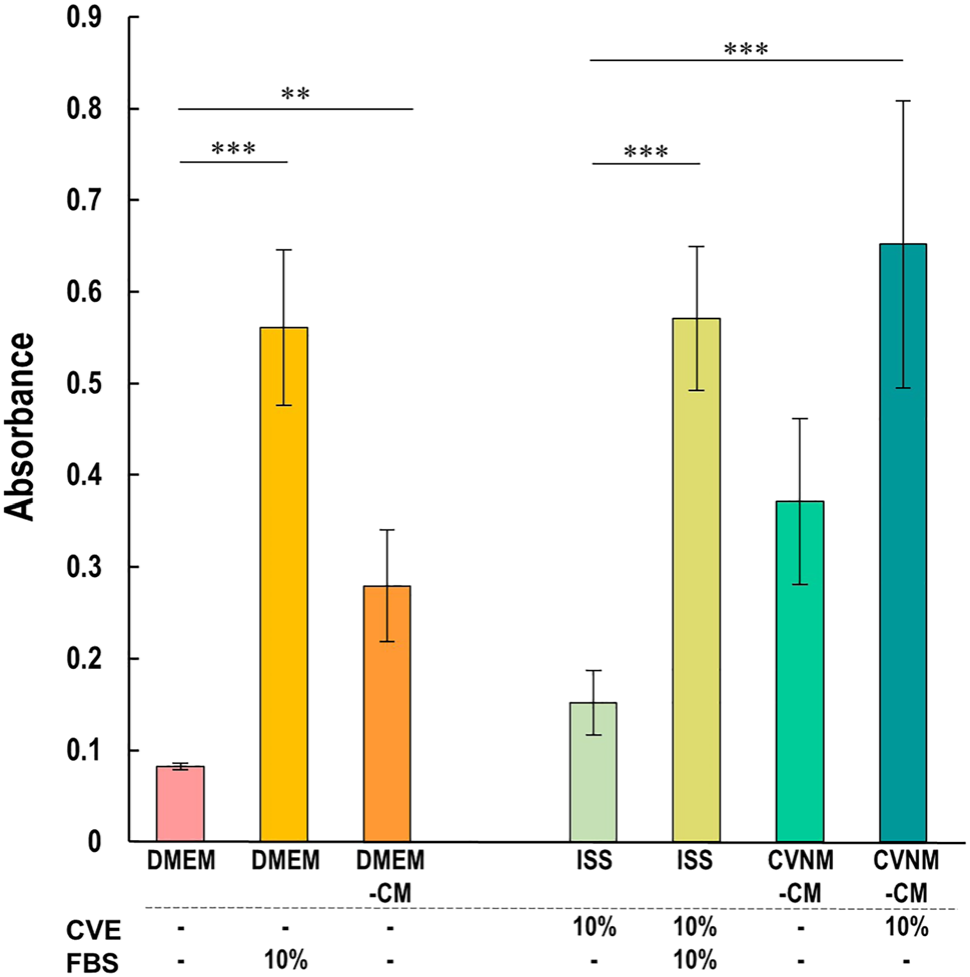
Proliferation of bovine myoblasts cultured with DMEM and CVNM for 2 days. The following media were used: DMEM, DMEM with 10% FBS, DMEM-CM, ISS with 10% CVE (10% CVNM; control for CVE-based media), 10% CVNM with 10% FBS, CVNM-CM, and CVNM-CM with 10% CVE. Data are presented as the mean ± standard deviation (n = 3). Statistical analysis was performed on the data set in Supplementary Fig. S1. ***P < 0.001, **P < 0.01*.* CVE, *Chlorella vulgaris* extract; CVNM, *C. vulgaris*-derived nutrient media; DMEM, Dulbecco's modified Eagle's medium; DMEM-CM, DMEM-based conditioned media; FBS, fetal bovine serum; ISS, inorganic salt solution.

Bovine myoblasts, collected from bovine muscle tissues as previously reported^10,13^, were cultured with the CVNM-based conditioned media from RL34 cells (CVNM-CM). Since adding more than 15% CVE to CVNM-CM decreased the proliferative capacity, 10% CVE was added to supplement the nutrients consumed by the RL34 cells (Supplementary Fig. S1). Therefore, the CVNM optimized for bovine myoblasts was ISS containing 10% CVE (10% CVNM). On day 2, the results of the XTT assay showed a comparable metabolic activity in CVNM-CM containing 10% CVE to that in 10% CVNM with 10% FBS (Fig. 7). For reference, proliferation assays were performed using DMEM, DMEM with 10% FBS, and the DMEM-based conditioned media from RL34 cells (DMEM-CM).

The proliferated cells were fixed after 48 h of culture and stained with MyoD, a marker of myogenic cells, to confirm their myogenicity. The cells were then imaged and the percentage of MyoD-positive cells to Hoechst-stained cells was determined. There were no significant differences between the different culture conditions, with more than 98% positive for MyoD, indicating that most of the proliferated cells were myoblasts (Supplementary Fig. S2a). However, there were discrepancies between the counts obtained for the Hoechst-stained cells (Supplementary Fig. S2b) and the estimated counts of the number of viable cells obtained according to the metabolic activity using the XTT assay (Fig. 7). Measurement by the XTT assay showed higher overall absorbance in the CVE-based media compared to DMEM-based media. It is possible that CVE increased the overall metabolic activity in the CVE-based medium, and that the growth rate cannot be accurately measured in comparison to DMEM-based media. For these reasons, statistical analysis was performed for each of the DMEM- and CVE-based media for the data sets of the conditions as shown in Supplementary Fig. S1. Figure 7 provides an excerpt of the results of representative conditions that were subsequently followed.

Further, the gene expression levels of the satellite cell marker *PAX7* and the myogenic determinant genes, *MYOD* and *MYF5,* were analyzed by real-time quantitative PCR (RT-qPCR) before and after 48 h of culture in DMEM with 10% FBS and CVNM-CM with 10% CVE. The expression of *PAX7* mRNA was not significantly different before and after incubation in both media. In addition, the expression of *MYOD* and *MYF5* mRNA were increased in DMEM with 10% FBS, but not significantly changed in CVNM-CM with 10% CVE (Supplementary Fig. S3).

### Scaling up of the culture system

**Figure 8:**
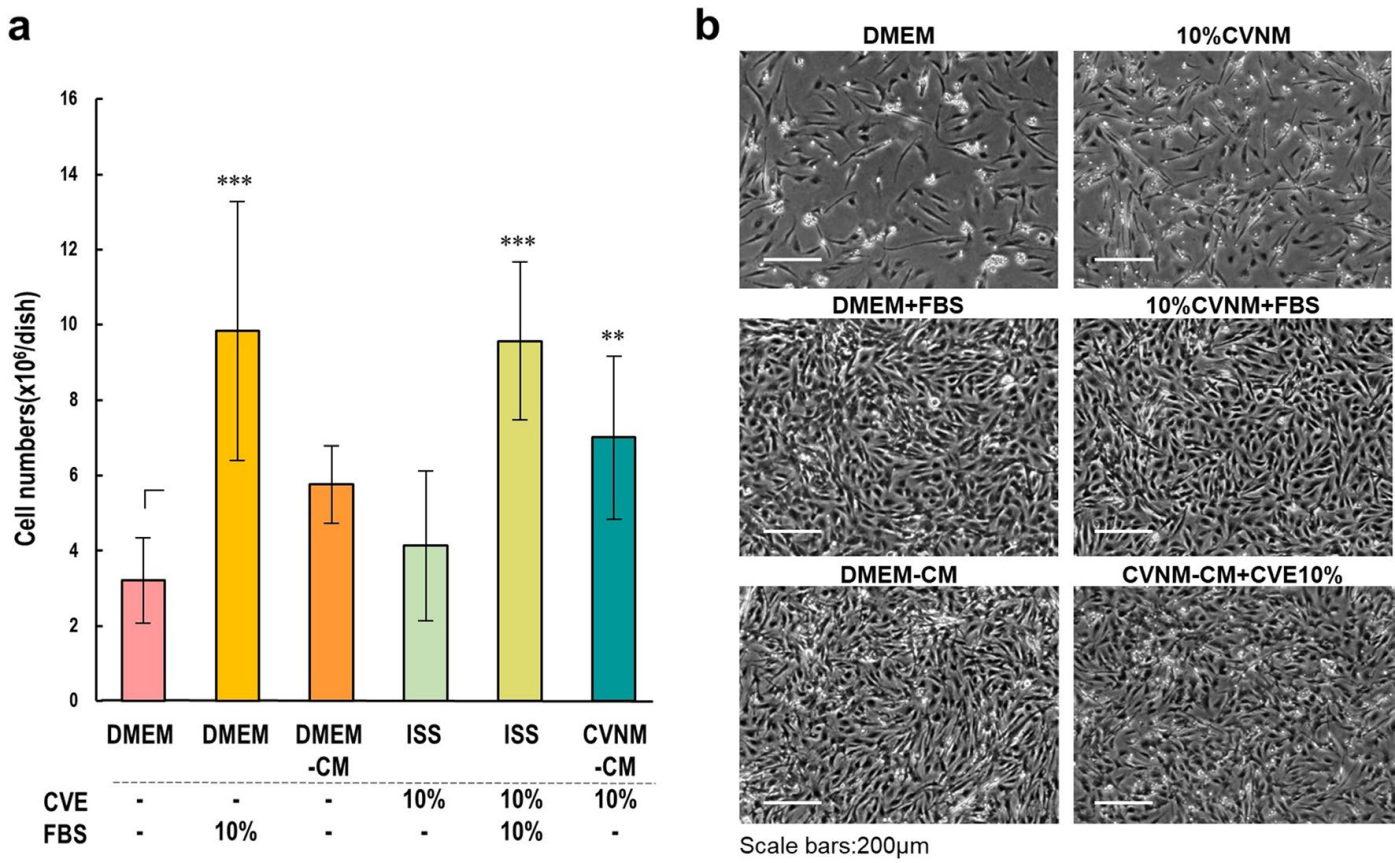
Scaling up of culture system (**a**) Cell numbers of primary bovine myoblasts cultured in 100 mm dish for 48 h. The following media were used: DMEM, DMEM with 10% FBS, DMEM-CM, ISS with 10% CVE, 10% CVNM with 10% FBS, and CVNM-CM with 10% CVE. Data are presented as the mean ± standard deviation (n = 5–7). ***P < 0.001, **P < 0.01. (**b**) Images of primary bovine myoblasts under the different media conditions for 48 h. CVE, *Chlorella vulgaris* extract; CVNM, *C. vulgaris*-derived nutrient media; CVNM-CM, CVNM-based conditioned media; DMEM, Dulbecco's modified Eagle's medium; DMEM-CM, DMEM-based conditioned media; FBS, fetal bovine serum; ISS, inorganic salt solution.

We attempted to scale-up the process for application to cultured meat production. Bovine myoblasts (5 × 10^4^ cells/cm^2^) were seeded in a 100 mm dish with DMEM with 10% FBS. After adhering, the cells were cultured under the different culture conditions as described above. In this experiment, half of the medium was renewed the next day. Figure 8 shows the number of cells per dish (Fig. 8a) and images after 48 h of culture under each condition (Fig. 8b). Although the growth rate was lower than in basal media containing 10% FBS, bovine myoblasts were found to grow in the medium containing microalga-derived nutrients and cell-secreted growth factors (CVNM-CM).

### Growth factor contained in CVNM-CM

The following factors have been reported to promote the proliferation of myoblasts: fibroblast growth factor-2 (FGF-2)^14–18^, insulin-like growth factor-1 (IGF-1)^15,19,20^, platelet-derived growth factor (PDGF)^15,18,21^, and leukemia inhibitory factor (LIF)^22^. Since RL34 cells are epithelial cells derived from rat liver, we focused on IGF-1, which is synthesized primarily by the liver (> 70%)^23,24^. The concentration of IGF-1 measured by enzyme-linked immunosorbent assay (ELISA) at 24 h of cultivation was 54.54 ± 5.68 and 48.58 ± 22.44 pg/mL in DMEM-CM and CVNM-CM, respectively (n = 3, mean ± SD). Since IGF-1 conserved 70% of amino acid sequences with IGF-2, we also measured IGF-2 concentrations. These were found to be 35.66 ± 6.28 and 124.13 ± 19 0.08 ng/mL in DMEM-CM and CVNM-CM, respectively (n = 4, mean ± SD), which are orders of magnitude higher than the concentration of IGF-1. Thus, we can assume that IGF-2 was one of the key factors that promoted the proliferation of bovine myoblasts. Further, the concentration of IGF-2 was significantly higher in CVNM-CM than in DMEM-CM (by Mann–Whitney *U* test, *P* < *0.05*).

## Discussion

CVE has both positive effects that promote cell survival and proliferation^9,10,25,26^, and negative effects that cause cell injury and death^27,28^. Increasing the CVE concentration in CVNM resulted in cell damage and the eventual death of almost all cells (Fig. 3a). The results also showed that the proliferative effect of CVE on bovine myoblasts was limited (Fig. 8). To maximize the effectiveness of CVNM-CM in maintaining cell survival and promoting cell proliferation, CVNM needed to contain at least sufficient nutrients, including the maximum possible amounts of CVE. However, the substances toxic to the cells were not identified in this study; hence, they could not be removed or neutralized. Identifying these substances is imperative in developing methods to reduce the cytotoxicity of CVE, allowing the production of nutrient-rich CVNM containing sufficient CVE. This would improve the survival environment of RL34 cells, allowing them to secrete high levels of growth factors, thereby increasing their proliferative potential. Furthermore, several studies have reported that microalgae extracts have a proliferative effect^25,26^ and that increasing their content might have a synergistic effect with the growth factors secreted by RL34 cells.

In this study, we initially focused on the extraction method of microalgal nutrients. There were differences in the composition of nutrients between the CVEs subjected to acid hydrolysis and ultrasonic fractionation (Fig. 2a,b). While ultrasonic fractionation yielded no glucose, it was able to extract glutamine and asparagine, which was not possible with acid hydrolysis. Glutamine is the most abundant amino acid in plasma and a principal source of cellular energy; it is generally present in greater amounts than other amino acids in most standard culture media. Although it can be substituted with glutamic acid^9^, this may cause cytotoxicity in media containing no glutamine. Since asparagine, a non-essential amino acid (NEAA), is synthesized intracellularly, some of the standard culture media, such as DMEM, does not contain it. While many cells do not synthesize sufficient amounts of NEAAs in vitro, this is inconsequential because they are routinely cultured with serum which contains free amino acids^11,29^. Nevertheless, adding asparagine is necessary in serum-free culture if the cell lines require it. In normal hepatocytes, glutamine uptake has been known to be mediated by System N, a Na^+^-dependent amino acid transporter, and asparagine plays a predominant role in this activation compared to other amino acids^30^. Similarly, asparagine stimulates carboxylase synthesis more robustly than other amino acids in rat hepatocytes^31^. We hypothesized that the addition of UFE may have acted to compensate for the lack of amino acids in AHE in the RL34 cell culture (Fig. 4a,b). In fact, when RL34 cells were cultured in a medium containing only AHE and supplemented with glutamine or asparagine, there was an increase in the number of viable cells. The concentration of the added glutamine was relatively lower than that in DMEM (584 mg/L) but was comparable to that in 15% CVNM, which contained 7.5% UFE (5.41 ± 0.76 mg/L; final concentration calculated from Fig. 2b). This suggests that amino acid content should be balanced and that the excess or deficiency of certain amino acids may cause ‘amino acid imbalance’ which may suppress cell viability^32^.

The osmolality of most standard media is based on the body fluid, which is generally 290 ± 30 mOsm/kg. For example, the osmolality of standard DMEM is between 330 and 360 mOsm/kg. However, since the optimal osmolality differs between tissue types and animal species, it is necessary to determine the appropriate osmolality for each cell culture. For example, there are reports that high osmolality is associated with a significant reduction in cell growth rate in fed-batch culturing of Chinese hamster ovary (CHO) cells for recombinant protein production^12,33^. Our results revealed that, in RL34 cells, 15% CVNM showed the highest cell viability under a pressure of 260–300 mOsm/kg (Fig. 5b). When RL34 cells are cultured in CVNM, the osmolality may increase slightly owing to evaporation of the medium and production of metabolites. Furthermore, since 10% CVE is further added to compensate for the nutrients consumed by RL34 cells, the osmolality would increase even more. Therefore, the osmolality of CVNM should be kept low to culture bovine myoblasts within the physiologically suitable osmolality range. From this perspective, RL34 cells serve as suitable provider cells in this system due to their high survival rates at low osmolalities.

Studies report that a polypeptide with somatomedin (its three forms include IGF-1, IGF-2, and vitronectin)-like activity is present in the CM of rat liver cells and that it promotes the proliferation of chicken embryo fibroblasts^34–37^. The most abundant IGF circulating in human blood is IGF-2, which is ~ 700 ng/mL in adults, three times the amount of IGF-1^38^. FBS, which is used for cell culture, contains 500–900 ng/mL of IGF protein^39^. Our results indicate that the concentration of IGF protein in the CM was comparable to that of 10% FBS in DMEM. In the field of regenerative medicine research, the potency of stem cell therapy is owed to the paracrine effect of the secreted factors^40–42^. Several studies report that the CM collected from mesenchymal stem cells, which contain multiple growth factors and cytokines, has the ability to regenerate tissue^43,44^. They work synergistically and show regenerative potential in the treatment of various diseases^45^. Furthermore, considering that FBS contains 143 different proteins and several of them are growth-related^46^, it is highly likely that there are other growth factors in CVNM-CM whose synergistic effect may have contributed to the improved cell proliferation. Since the type and amount of growth factors secreted in CM vary by the type of provider cell and culture medium^41,45^, the key to practical application is to find the CVNM-CM which most promotes myoblast proliferation.

Another consideration is the maintenance of provider cells in serum-free media. In this study, RL34 cells proliferated in serum-free conditions and stopped increasing on day 3 when cultured using CVNM for the production of CM; the same was true when DMEM was used (Fig. 6). The reason for this trend may be that the RL34 cells were over-confluent in DMEM and had started to acclimate in both conditions. Our results showed that the cells survived serum-free media for at least 4 days during which the supernatant could be collected. CVNM-CM is sufficient for cell culture when all factors are balanced according to the favorable conditions for the cells and when the cells are acclimated to them.

There are several obstacles hindering the use of CM for cultured meat production. Myoblasts were shown to proliferate in the CM produced by fibroblasts^47,48^; thus, the idea of using CM for cultured meat production was proposed^49^. Our results showed that, compared to FBS-containing media, cells cultured in CM had reduced proliferative capacity (Fig. 8). This might be because the factors present in the CM are not exactly the same as those in FBS. FBS plays many roles, including promoting cell proliferation, assisting the adherence of cells to scaffolds, reducing shear stress, and neutralizing toxic components^11,50^. Therefore, our results indicate that the CM of RL34 cells could replace only some of the capabilities that FBS possesses. IGF-1 and IGF-2 were found to be secreted into the CM by RL34 cells; however, other factors, such as growth, adhesion, and toxicity neutralizing factors, were not examined. The limited effect of CVNM-CM on bovine myoblast proliferation compared to FBS-containing media suggests that CVNM-CM does not contain these factors in sufficient quantities. This also explains the difference in *MYOD* and *MYF5* mRNA expressions of bovine myoblasts cultured in DMEM with 10% FBS and in CVNM-CM with 10% CVE (Supplementary Fig. S3).

Another challenge is that currently, we have not yet established a method for differentiating myoblasts into myotubes by using CVNM-CM. Conventionally, myoblasts are cultured in serum-containing media, and their differentiation into myotubes is induced when the quantity of serum in the medium is reduced, a state called ‘serum starvation.’ There are several reports of chemically defined media for cultured meat production, each designed for myogenic cell proliferation and differentiation. The proliferation media were based on commercially available basal media with added purified proteins that promote proliferation and adhesion^51,52^. The formulation of those proteins was adjusted to promote myogenic cell differentiation^53^. Recently, a medium for differentiating bovine myogenic cells was developed by adding ligands for receptors whose expression increased when myogenic cells differentiated in serum-free medium^54^. With these reports as reference, adding factors that are deficient in the CVNM-CM is one way to address this challenge. For increased sustainability, the next step is to find provider cells that secrete factors not present in the CM of RL34 cells, and that are capable of compensating for the functions of proliferation or differentiation. To that end, mixing appropriate proportions of multiple provider cell CMs would be as effective and efficient as chemically defined media.

An advantage of using CVNM is that the microalgal extract has many functional properties besides nutrients. Hydrolyzed extracts of plants have been widely used in the production of biopharmaceuticals as an additive in animal cell culture media for many years. In particular, soy hydrolysates have been well-studied in the trend towards eliminating animal-derived components in the production of proteins using CHO cells. These hydrolysates are known for their ability to promote cell growth and increase protein production capacity besides providing nutrients such as amino acids and vitamins to the medium. These effects are considered to be conferred by the physiological activities of peptides, the main components of hydrolysates, such as antioxidant, immunomodulatory, and protease inhibitory effects^55^. Moreover, polysaccharides extracted from *C. vulgaris* possess antioxidant properties. The activity level varies depending on the extraction method, but UFEs reportedly exhibit higher antioxidant activity compared to extracts from other methods^56^. In our study, the bovine myoblasts cultured in 10% or 15% CVNM alone showed significantly higher values in the XTT assay compared to those cultured in DMEM alone (by Welch’s *t* test, *P* < 0.05). Although there might be artifacts in the XTT assay as mentioned above, owing to the high metabolic activity of CVE, there was also a trend towards proliferation in the cell counts (DMEM vs 10% CVNM); however, this was not statistically significant. As 10% CVNM had a lower nutrient than DMEM, it did not show high proliferative potential. Nevertheless, CVNM by itself may have proliferative activity. Moreover, given the higher content of IGF-2 in CVNM-CM compared to DMEM-CM, culturing cells in CVNM may be an advantage for provider cell cultures. An in-depth study should be conducted to assess the effects of microalgal extract on cell proliferation and metabolic activity.

To the best of our knowledge, this is the first study to report that myoblasts can proliferate using microalga-derived nutrients and cell-secreted factors as alternatives to grain-derived nutrients and animal serum, respectively. Further research should be conducted to identify the factors in CVNM-CM that influence metabolic activity, cell proliferation, and cell cytotoxicity, as well as to develop technologies capable of adjusting them. This would enable their applications to be expanded from research to industry. We believe that the technology we have developed for sustainable culture medium production will contribute not only to cultured meat production, but also to other industries based on cell culture technology in the future.

## Methods

### Isolation and culture of primary myoblasts from bovine tissue

Myoblasts were isolated from the cheek skeletal muscle tissues of Japanese Black cattle, provided by a local slaughterhouse (Tokyo Shibaura Zoki, Tokyo, Japan), as previously described^10,13^. Briefly, bovine muscle tissue from the day of slaughter was sterilized and minced into small pieces. The minced sample was digested using 1 mg/mL of pronase (from *Streptomyces griseus*, Sigma-Aldrich, St. Louis, MO) in Hanks’ balanced salt solution, followed by incubation at 37 ℃ with agitation at 100 rpm for 60 min. An equal volume of DMEM (high glucose) with L-glutamine, phenol red, and sodium pyruvate (FUJIFILM Wako Pure Chemical, Osaka, Japan), supplemented with 10% FBS was then added to suspension and mechanically disrupted using a measuring pipette. The suspension sample was filtered using a 40 µm cell strainer and centrifuged at 1000×*g* for 10 min to collect the myoblasts. The supernatant was discarded and the precipitate was dissolved in growth media consisting of DMEM with 10% FBS, 1% penicillin-streptomycin-amphotericin B, and 10 ng/mL FGF-2 (Kaken Pharmaceutical, Tokyo, Japan). To exclude the non-myogenic cells that adhered initially, such as fibroblasts, the cell suspension was incubated in a non-coated cell culture dish at 37 °C for 1 h and then the supernatant was seeded (diluted to 1:5) in cell culture dishes coated with recombinant laminin-511 E8 fragment (Easy iMatrix-511, Nippi, Tokyo, Japan). After 7 days, the obtained primary bovine cells were passaged in growth media and used for experiments up to three passages.

### Culturing of *C. vulgaris* and nutrient extraction

*C. vulgaris* (NIES-2170) was obtained from the National Institute for Environmental Studies (Ibaraki, Japan) and cultured in Gamborg’s B5 medium (Fujifilm Wako Pure Chemical) mixed with 1 g/L fertilizer (HYPONeX Japan, Osaka, Japan), as previously described^10^. *C. vulgaris* biomass was then harvested by centrifugation and resuspended in pure water; this process was repeated twice to wash out the culture medium. The precipitate was then lyophilized using a freeze drier (FDU-1200, Tokyo Rikakikai, Tokyo, Japan) to obtain powdered *C. vulgaris*.

For the AHE, the powder was dissolved in 0.25, 0.5, or 1 N hydrochloric acid at a concentration of 50 g/L, incubated at 100 °C for 24 h, and neutralized with sodium hydroxide. For the UFE, the powder was dissolved in distilled water at a concentration of 50 or 100 g/L and ultrasonically crushed in ice cold water for 10 min using an ultrasonic crusher (Bioruptor UCD-200TM, Cosmo Bio, Tokyo, Japan) at 200 W. After extracting the nutrients, the suspension was centrifuged at 15,000×*g* for 10 min and the resultant residue was removed. The supernatant was then sterilized by filtration through a 0.22 μm pore polyvinylidene fluoride filter and used for the experiment as the CVE. Analysis of the nutrient content and osmolality of the extracts was outsourced to the clinical laboratory company SRL Inc. (Tokyo, Japan). UV-hexokinase method and liquid chromatography-mass spectrometry was used for measuring the glucose and amino acid content, respectively, while freezing point depression method was used to measure the osmolality. The pH was measured using a LAQUAtwin pH meter (HORIBA, Kyoto, Japan) after the medium alone (without cells) was placed in a CO_2_ incubator for 24 h to stabilize the values.

### Preparation of CVNM and CM

First, a preliminary experiment was conducted to determine the optimal conditions. To achieve this, concentrated ISS was prepared. The ISS composition is described in Table 1. Different compositions of CVE (0.25–1 N AHE, mixture of equal volumes of 0.25 or 0.5 N AHE and 100 g/L UFE) extracted by the above methods were added at concentrations of 0, 2, 5, 10, 15, 20, and 25% (V/V), and the osmolality and concentration of the ISS were equalized with sodium chloride and water.

RL34 cells, a rat liver epithelial cell line, were maintained in DMEM supplemented with 10% FBS and 1% penicillin-streptmycin (P/S). Prior to the experiments, the cells were seeded at a density of 1 × 10^5^ cells/cm^2^ in growth media and permitted to adhere to the culture dish. The next day, after reaching 85–95% confluency, the cells were washed with phosphate buffer saline (PBS) and incubated in either DMEM or ISS containing various concentrations of CVE.

Based on the results of the preliminary experiments, 15% CVE (mixture of equal volumes of 0.5 N AHE and UFE) added to ISS (hereafter referred to as CVNM) was used for the preparation of CM. To collect the CM, RL34 cells were seeded in 35 mm or 100 mm dishes at a density of 1 × 10^5^ cells/cm^2^ and permitted to adhere for 24 h. The media were removed the next day, following which the cells were washed with PBS and incubated with either DMEM or CVNM. The CM was collected at 24 h after incubation and frozen at − 30 °C, following which it was thawed and centrifuged at 15,000 × *g* for 10 min to remove any residual dead RL34 cells.

### Viability and proliferation assay (XTT Assay)

The viability of RL34 cells and proliferation rates of bovine myoblasts were evaluated using an XTT assay kit (Biological Industries, Beit-Haemek, Israel), as described previously^10^. Briefly, RL34 cells were seeded in a 96-well plate at 1 × 10^5^ cells/cm^2^ (85–95% confluency) along with DMEM supplemented with 10% FBS and 1% P/S, and permitted to adhere for 24 h. The media were then removed and the cells were washed with PBS; thereafter, ISS containing different concentrations of each CVE was added to the wells. In the amino acid supplementation test, RL34 cells were cultured in ISS containing 5% of 0.5 N AHE with/without the addition of l-glutamine solution (FUJIFILM Wako Pure Chemical) or l-asparagine (MP Biomedicals, Inc., Irvine, CA) at concentrations of 1–10 mg/L and 5–20 mg/L, respectively. After 24 h, the medium was removed to avoid the absorbance effect of CVE, after which 100 µL of DMEM and 50 µL of XTT reaction solution were added to each well. After incubation at 37 °C for 2 h, the metabolic activity of the cells was evaluated by subtracting the optical density measured at 650 nm from that measured at 450 nm. To evaluate the potential proliferation of bovine myoblasts, the cells (1 × 10^5^ cells/cm^2^, 30–40% confluency) were cultured using the different medium compositions as shown in Supplementary Fig. S1 other than those described above. Further, 10% CVE was added to CVNM-CM to compensate for the nutrients consumed by RL34 cells. Therefore, 10% CVE (mixture of equal volumes of AHE and UFE) added to ISS was used as the comparison for CVE-based media.

It should be noted that RL34 cells and bovine myoblasts clearly show different confluency given the difference in cell size and adhesion morphology, even when seeded at the same density.

### Cell counting

Cells were trypsinized and resuspended in DMEM containing 10% FBS and 1% P/S. After staining with trypan blue, the total viable cell number was determined using an automated cell counter (Countess, Thermo Fisher Scientific, Waltham, MA).

For long-term cell viability assay, RL34 cells were seeded in a 24-well plate at 1 × 10^5^ cells/cm^2^ using DMEM with 10% FBS and 1% P/S, and permitted to adhere for 24 h. The media were then removed and the cells were washed with PBS, incubated with the different media compositions. Two replicate wells were included for each composition and the media were changed every day. The number of cells in each well was counted daily up to day 4.

For the bovine primary cell proliferation assay, the cells were seeded in 100 mm dishes at 5.5 × 10^4^ cells/cm^2^ using DMEM with 10% FBS and 1% P/S, and permitted to adhere for 24 h. Thereafter, the media were removed and the cells were washed with PBS and incubated with the different media compositions. After 24 h, half of the media were removed and replaced with fresh media. The cells were counted at 48 h of culture as described above.

### IGF-1 and IGF-2 measurement

The concentrations of IGF-1 and IGF-2 in DMEM-CM and CVNM-CM were measured using a Mouse/Rat IGF-1 Quantikine ELISA Kit (R&D Systems, Minneapolis, MN) and Mouse/Rat/Porcine/Canine IGF2 Quantikine ELISA Kit (R&D Systems) according to the manufacturer’s instructions.

### Statistical analysis

Each experiment was performed in duplicates or triplicates and all data were presented as means ± SD of at least three independent experiments. Statistical analysis was performed using R software (version 4.1.3). Welch’s *t* test and Mann–Whitney *U* test were used to compare data between the two groups. Multiple comparisons were conducted using analysis of variance (ANOVA) followed by Tukey’s honest significance test against every mean or Dunnett’s test against the control mean as appropriate. Differences were considered significant at *P* < 0.05.

## Supplementary Information

Supplementary Information.

## Data Availability

The datasets generated in this study are available from the corresponding author on reasonable request.
