# Supplementary material for: Development of serum-free and grain-derived-nutrient-free medium using microalga-derived nutrients and mammalian cell-secreted growth factors for sustainable cultured meat production
Source: Sci Rep. 2023 Jan 10;13:498. doi: 10.1038/s41598-023-27629-w (PMC9832167; doi:10.1038/s41598-023-27629-w)
Supplement: Supplementary file 1 — Supplementary Information. [file 41598_2023_27629_MOESM1_ESM.pdf]

Supplementary Method

Immunofluorescence staining

Bovine myoblasts (1×10^5 cells/cm^2) were cultured using the different media compositions as shown in Supplementary Figure 1 in a 96-well plate. After 48 h, the cells were fixed with 4% paraformaldehyde and permeabilized with 0.25% Triton-X solution. After blocking with 2% bovine serum albumin (BSA), the cells were incubated with the primary mouse monoclonal anti-MyoD antibody (1:1000) (sc377460, Santa Cruz Biotechnology, Dallas, TX) at 4 °C overnight. Subsequently, the cells were incubated with Alexa Fluor-conjugated secondary antibody (1:500) (Thermo Fisher Scientific, Waltham, MA) at room temperature for 2 h. The cell nuclei were then stained with Hoechst 33342 (1:500) (Thermo Fisher Scientific). In between each step, the cells were washed three times with PBS to minimize cross-influences. Images were captured using an ECLIPSE Ti microscope (Nikon, Tokyo, Japan) and the number of nuclei and positive cells were counted using the ImageJ (1.53e) program. The percentages of MyoD-positive cells to total cells were calculated by counting two selected fields per well for each media.

RNA analysis

Total RNA was isolated from bovine primary cells before passage (pre-culture) and after 48 h culture under CVNM-CM using NucleoSpin® RNA Plus (TAKARA BIO INC., Shiga, Japan). cDNA was generated using SuperScript® III Reverse Transcriptase (Thermo Fisher scientific, Waltham, MA, USA). The abundance of transcripts was assessed by real-time, quantitative PCR (qPCR) analysis using the Fast SYBR® Green Master Mix (Thermo Fisher scientific, Waltham, MA, USA) and gene-specific primer pairs listed below. The qPCR was performed with an Applied Biosystems ViiATM7 real-time PCR system and analysis was performed using the $2^{-\Delta\Delta Ct}$ method. The forward (F) and reverse (R) primers used were as follows:

	Forward	Reverse
PAX7	5'-AGTGAGTTCGATTAGCCGAGTG-3'	5'-TGCTGTGCTTGGCTTTCTTC-3'
MYOD	5'- ACGTCTAGCAACCCAAACCAG-3'	5'- TGCAGGCCTTCGATATAGCG-3'
MYF5	5'- AAGTTGCTCTGATGGCATGC-3'	5'- AGACGCTGTCAAAACTGCTG-3'
ACTB	5'- TGCGGCATTCACGAAACTAC-3'	5'- TGTTGGCGTAGAGGTCCTTG-3'
B2M	5'-TCAAGACACCCACCAGAAGATG-3'	5'-TGAAAGACAGGTCTGACTGCTC-3'

All the expression levels were normalized to β-Actin (ACTB) and β-2-Microglobulin (B2M).The bars show the fold change relative to the mean of the pre-culture sample for each gene expression level.

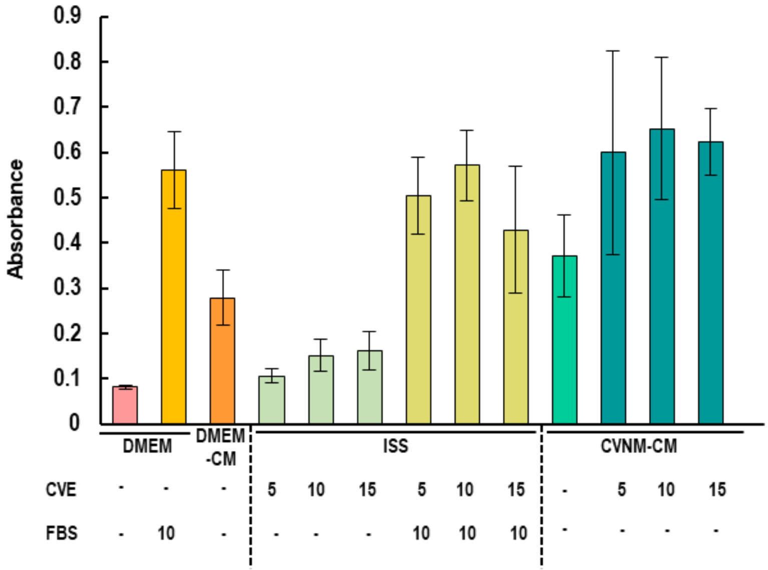

Supplementary Figure 1

Proliferation of bovine myoblasts cultured with DMEM-based media and CVE-based media for 2 days. Data are presented as the mean ± standard deviation (n = 3). CVE, *Chlorella vulgaris* extract; CVNM-CM, *C. vulgaris*-derived nutrient media-based conditioned media; DMEM, Dulbecco's modified Eagle's medium; DMEM-CM, DMEM-based conditioned media; FBS, fetal bovine serum; ISS, inorganic salt solution

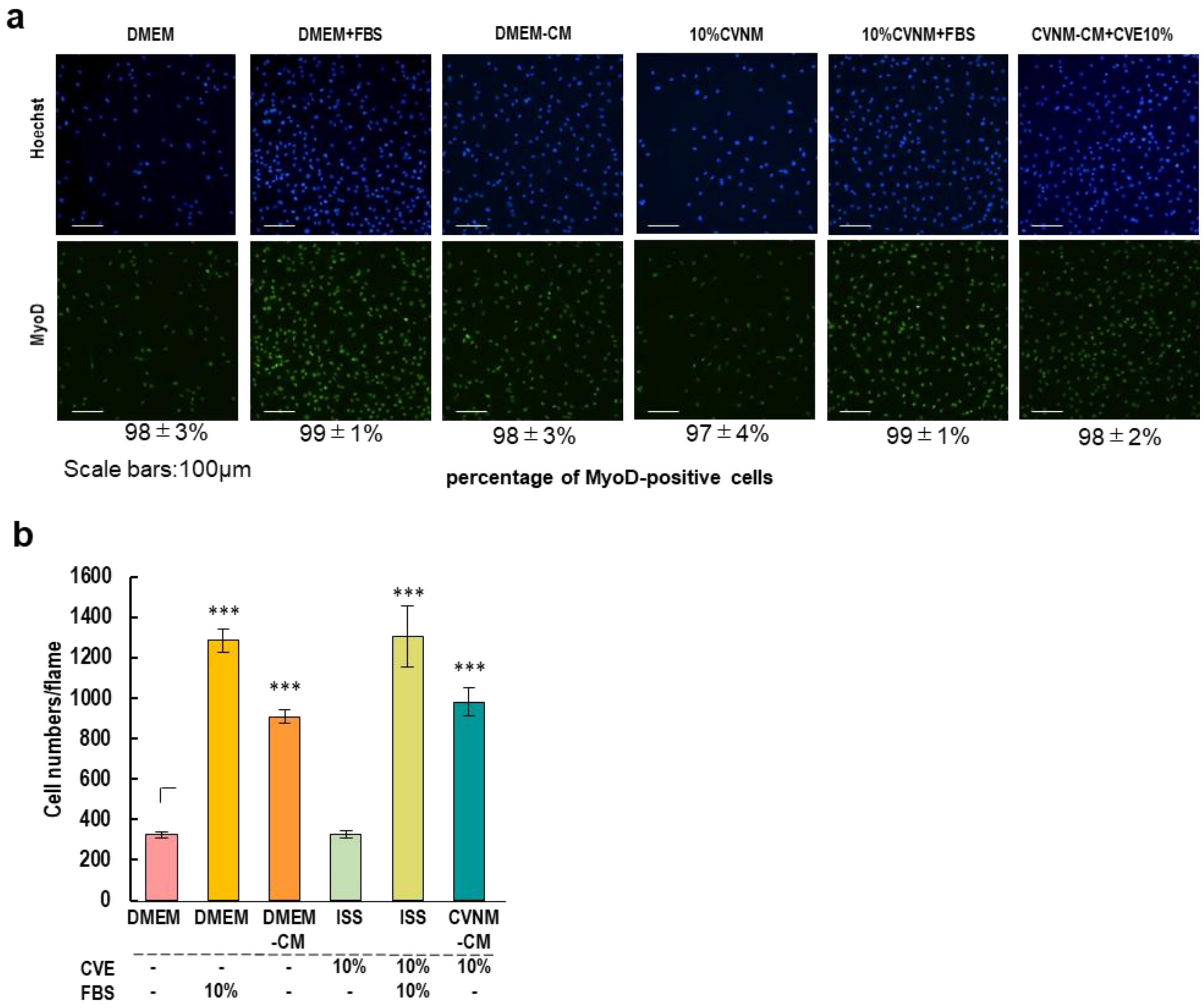

Supplementary Figure 2

(**a**) Images of immunofluorescence for the myogenic protein MyoD (green) under each condition for 48 h. Hoechst (blue) was used as a nuclear stain. The percentage of MyoD-positive cells are shown below the image. Data are represented as the mean ± standard deviation (n = 3). (**b**) Cell numbers measured by Image-based manual cell counting. Hoechst (blue)-stained cells were counted under each condition of two selected frames in each well. Data are presented as the mean ± standard deviation (n = 3). ****P<0.001*. CVE, *Chlorella vulgaris* extract; CVNM, *C. vulgaris*-derived nutrient media; CVNM-CM, CVNM-based conditioned media; DMEM, Dulbecco's modified Eagle's medium; DMEM-CM, DMEM-based conditioned media; FBS, fetal bovine serum; ISS, inorganic salt solution

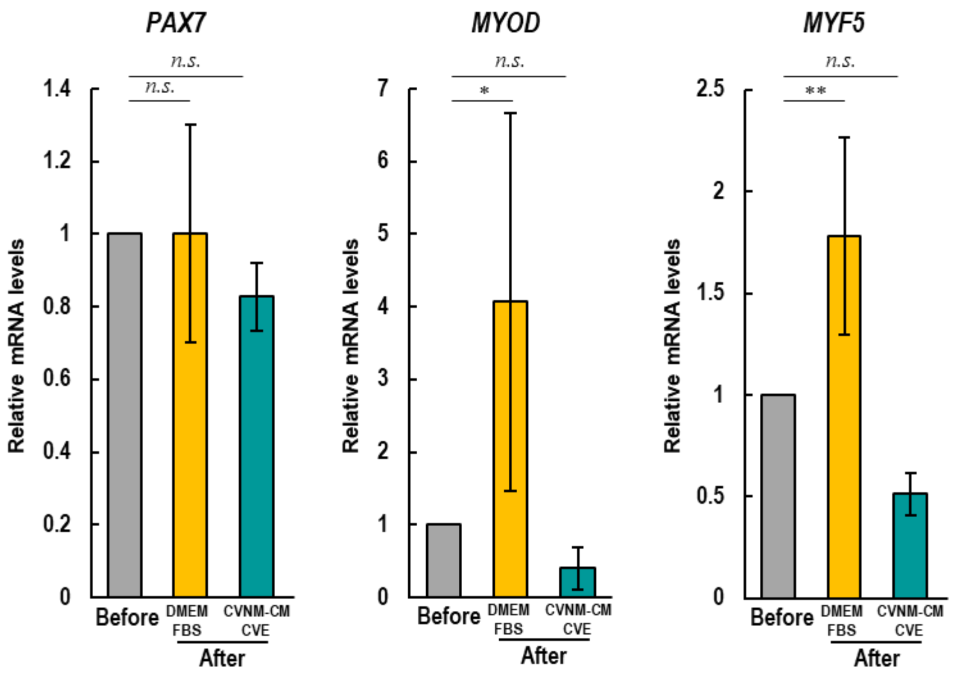

Supplementary Figure 3

RNA analysis of bovine myoblasts. The mRNA expressions of the myogenic genes (*PAX7, MYOD, MYF5*) after 48 h cultured with DMEM with 10% FBS and CVNM-CM with 10% CVE relative to before culture. Data are presented as the mean ± standard deviation (n = 4). ***P<0.01*, **P<0.05.* , *n.s., not significant*. CVE, *Chlorella vulgari*s extract; CVNM-CM, *C. vulgari*s-derived nutrient media-based conditioned media; DMEM, Dulbecco's modified Eagle's medium; FBS, fetal bovine serum
